# Is Walking Capacity in Subjects with Multiple Sclerosis Primarily Related to Muscle Oxidative Capacity or Maximal Muscle Strength? A Pilot Study

**DOI:** 10.1155/2014/759030

**Published:** 2014-01-29

**Authors:** Dominique Hansen, Peter Feys, Inez Wens, Bert O. Eijnde

**Affiliations:** Rehabilitation Research Centre (REVAL), Biomedical Research Institute, Faculty of Medicine and Life Sciences, Hasselt University, Agoralaan Building A, 3590 Diepenbeek, Belgium

## Abstract

*Background and Purpose*. Walking capacity is reduced in subjects with multiple sclerosis (MS). To develop effective exercise interventions to enhance walking capacity, it is important to determine the impact of factors, modifiable by exercise intervention (maximal muscle strength versus muscle oxidative capacity), on walking capacity. The purpose of this pilot study is to discriminate between the impact of maximal muscle strength versus muscle oxidative capacity on walking capacity in subjects with MS. *Methods*. From 24 patients with MS, muscle oxidative capacity was determined by calculation of exercise-onset oxygen uptake kinetics (mean response time) during submaximal exercise bouts. Maximal muscle strength (isometric knee extension and flexion peak torque) was assessed on dynamometer. All subjects completed a 6-minute walking test. Relationships between walking capacity (as a percentage of normal value) and muscle strength (of knee flexors and extensors) versus muscle oxidative capacity were assessed in multivariate regression analyses. *Results*. The expanded disability status score (EDSS) showed a significant univariate correlation (*r* = −0.70, *P* < 0.004) with walking capacity. In multivariate regression analyses, EDSS and mean response time, but not muscle strength, were independently related to walking capacity (*P* < 0.05). *Conclusions*. Walking distance is, next to disability level and not taking neurologic symptoms/deficits into account, primarily related to muscle oxidative capacity in subjects with MS. Additional study is needed to further examine/verify these findings.

## 1. Introduction

The presence of a reduced exercise capacity in patients with multiple sclerosis (MS) has received considerable interest, even though the underlying mechanisms remain to be explored [1]. Walking tests are often used to determine exercise capacity in subjects with MS [2]. Subjects with MS often show an impaired walking capacity, which may relate to an increased risk for limited mobility [2–5].

A systematic review demonstrated the potential of exercise therapy to improve walking capacity in patients with MS [6]. Recent cross-sectional studies have examined in more detail which factors, that can be modified by exercise training, play a role in the walking capacity in subjects with MS [7–9]. Such studies are highly relevant since they might reveal what factors should be targeted through exercise intervention in these subjects when aiming to enhance walking capacity (muscle strength or muscle endurance capacity). From these studies it has been found that upper leg muscle strength is an important contributor to walking capacity in patients with MS [7, 9]. However, it is important to mention that walking capacity is also related to postural control, ataxia, spasticity, motor function, and other neurologic symptoms/deficits, but these factors are often not primarily targeted when patients with MS participate in exercise interventions.

Concluding that only muscle strength plays a role in walking capacity in patients with MS, and should be primarily targeted during exercise intervention, is too premature. First, these studies expressed the walking distance in meters for all patients. According to clinical guidelines, the percentage of predicted normal value of walking distance should be used when a single measurement of walking capacity is executed in a patient [10]. Subject characteristics, such as gender, age, body weight, and height should be taken into account when evaluating the outcome of a walking test: these factors could significantly affect walking capacity and thus limit the clinical interpretation. Second, these previous studies did not take into account the potential impact of muscle oxidative capacity on walking capacity.

Previous studies on muscle oxidative capacity in patients with MS found a lowered maximal oxygen uptake [1], smaller type 1 skeletal muscle fiber diameter, lower succinate dehydrogenase activity, and complex 1 deficiency in skeletal muscle mitochondria, as opposed to healthy counterparts [11–13]. This indicates a lowered muscle oxidative capacity in subjects with MS. However, it is difficult to assess muscle oxidative capacity: obtaining skeletal muscle biopsies would require invasive techniques and/or applying ^31^P magnetic resonance spectroscopy would be costly and technically demanding. A novel method to examine skeletal muscle oxidative capacity by noninvasive means is the assessment of exercise-onset oxygen uptake (VO_2_) kinetics [14]. It has been shown that VO_2_ kinetics are highly reproducible [15] and are faster in skeletal muscle with predominantly slow-twitch fibers and with increased activation of oxidative muscle enzymes [16, 17]. It is thus generally accepted that assessing exercise-onset VO_2_ kinetics is a sensitive tool for the specific evaluation of muscle oxidative capacity [18]. Intriguingly, exercise-onset VO_2_ kinetics are significantly slowed in subjects with MS [19]. Such impaired muscle oxidative capacity might thus interfere with walking capacity in subjects with MS.

The aim of this study is to examine the relation between walking capacity (as a percentage of normal value) and maximal muscle strength versus muscle oxidative capacity in subjects with MS. We hypothesized that walking capacity would primarily be related to muscle strength in persons with MS, when not taking neurologic symptoms/deficits (such as postural control, ataxia, spasticity, and motor function) that affect walking capacity into account [7, 9].

## 2. Methods

### 2.1. Subjects

**Figure 1:**
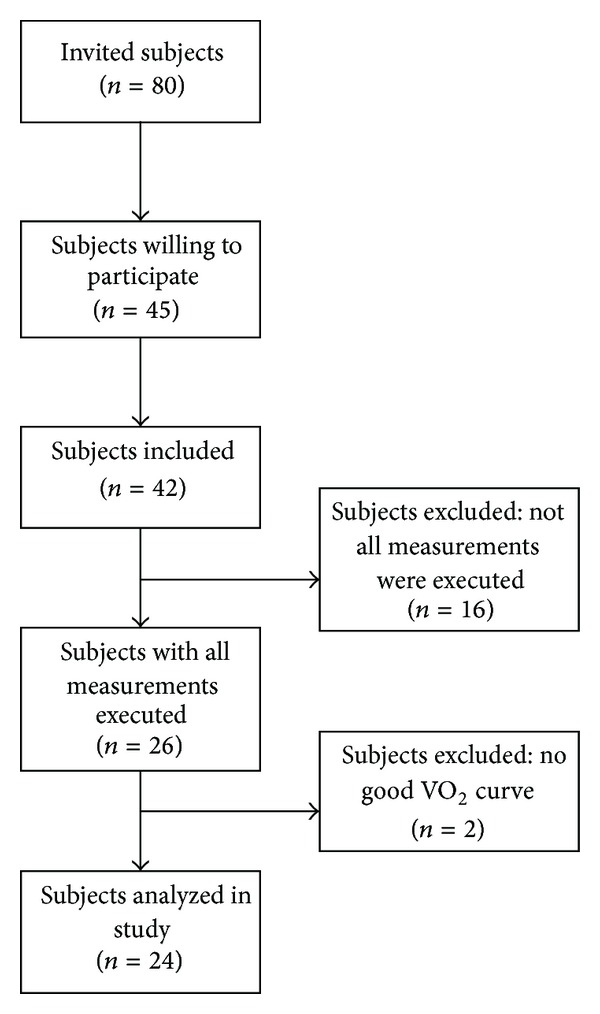
Study flowchart.

Twenty-four subjects with multiple sclerosis (MS) were selected to participate in this study (by publication of local advertisements and by contacting neurologists in a nearby MS clinic) (see Figure 1). These subjects participated in a larger ongoing study examining the impact of exercise intervention. Subjects had been diagnosed with MS for at least 12 months, did not experience >2 severe exacerbations in the last two years, were ambulatory with an expanded disability status scale (EDSS) score <6.0, and did not suffer from other chronic diseases. Subjects were informed about the nature and risks of the experimental procedures before their written informed consent was obtained. This study was approved by the medical ethical committee of Hasselt University.

### 2.2. Study Design

This was a cross-sectional study. Results were revealed to the subjects after completion of the study. Following EDSS determination [20] and medication intake screening by a neurologist, subjects underwent four experimental sessions (completed within two weeks). During the first experimental session body composition and physical activity were determined. In the second experimental session exercise-onset oxygen uptake (VO_2_) kinetics were determined during two subsequent submaximal endurance exercise bouts. During the third experimental session, a 6-minute walking test was executed. During the fourth experimental session, maximal muscle strength was assessed.

### 2.3. Descriptive Outcome Measures

#### 2.3.1. Body Composition

Following body weight (mechanical column scale with beam, Seca, Birmingham, UK) and length assessment, segmental and total-body adipose tissue mass and lean tissue mass were determined using whole body dual X-ray absorptiometry (DXA; Lunar DPXL, Wisconsin, USA) [21].

#### 2.3.2. Daily Physical Activity

Daily physical activity, related to sports and recreational activities, household activities, transportation, labor activities, and sitting time, was evaluated by the 13-item Physical Activity Scale for Individuals with Physical Disabilities (PASIPD) [22, 23]. From this questionnaire, the metabolic equivalent (MET) ∗ hours/week was calculated.

### 2.4. Experimental Outcome Measures

#### 2.4.1. Exercise Test

Subjects performed a submaximal exercise test on an electronically braked cycle ergometer (eBike Basic, General Electric GmbH, Bitz, Germany). It was decided to execute an exercise test on bike for the calculation of exercise-onset oxygen uptake (VO_2_) kinetics because neurologic symptoms/deficits that are often present in patients with MS are less interfering with proper calculation of exercise-onset VO_2_ than during walking. This methodology was thus chosen to obtain a more valid assessment of exercise-onset VO_2_ kinetics. In healthy subjects, between walking and cycling exercise-onset VO_2_ kinetics are not different when exercise intensities ≤ 90% of maximal heart rate are elicited [24]. During walking tests exercise intensities at about 67 ± 10 of estimated maximal heart rate are elicited in patients with MS [25]. Subjects were advised not to perform any exercise the day before or at the day of testing and only eat a light meal at least two hours prior to testing.

Pulmonary gas exchange was continuously measured breath-by-breath with a mass spectrometer and volume turbine system (Jaeger Oxycon, Erich Jaeger GmbH, Germany). During the exercise test, VO_2_ (mL/min) and expiratory volume (VE, L/min) were assessed breath-by-breath, after which these data were averaged every 10 seconds. Heart rate was continuously monitored by 12-lead ECG device. Predicted maximal heart rate was calculated by 220-age.

Following each exercise bout, capillary blood samples were obtained from the fingertip to analyze blood lactate concentrations (mmol/L), using a portable lactate analyzer (Accutrend Plus, Roche Diagnostics Limited, Sussex, UK) [26]. At the end of each exercise bout ratings of perceived exertion (RPE) was scored by the subject on a 6–20 Borg scale.

Subjects were seated on bike for three minutes to obtain resting data. Next, subjects were instructed to cycle at a rate of 70 rpm, against a resistance corresponding to 25% of predicted cycling power output (*W*_max⁡_), for six minutes [19]. After six minutes of cycling subjects remained seated on bike for additional six minutes, after which a second 6-minute exercise bout was initiated. Data collected during these two exercise bouts were averaged. All VO_2_ curves were analyzed in detail to verify that a VO_2_ steady-state was achieved within five minutes. Predicted *W*_max⁡_ was based on gender, age, body weight, and height and calculated by previously published formulae [27].

Exercise-onset VO_2_ kinetics were calculated algebraically and expressed as mean response time (MRT), as explained previously [19]. The two MRT's that were obtained from the two exercise bouts were averaged. A greater MRT indicates a worse skeletal muscle oxidative capacity.

#### 2.4.2. 6-Minute Walking Test

Subjects were instructed to walk for as much distance as they could within a 6-minute period [7, 28]. This test was applied according the instruction script of Goldman et al. [29]. Subjects walked, at maximal effort, back and forth in a 30-meter hallway turning round cones and were permitted to use their habitual assistive devices. Distances walked per minute and total distance were registered. At the end of the test, Borg ratings of perceived exertion were scored on a 6–20 scale. Normal walking distance was predicted by the following formulae [30]: for males, 6-minute walking distance (*m*) = (7.57∗height (in cm)) − (5.02∗age) − (1.76∗weight (in kg) − 309). For females, 6-minute walking distance (*m*) = (2.11∗height (in cm)) − (2.29∗weight (in kg) − (5.78∗age) + 667). Data from the walking test was expressed in a percentage of normal value.

#### 2.4.3. Muscle Strength Test

Maximal voluntary knee-extensor and flexor strength of both legs were evaluated on an isokinetic dynamometer (Biodex Medical Systems, system 3, Inc, Shirley, New York) [7]. After a 5-minute standardised warm-up on a cycle ergometer, strength tests were performed in a seated position on a backward inclined (5°) chair. The rotational axis of the dynamometer was aligned with the transverse knee-joint axis and connected to distal end of tibia by means of a length adjustable lever arm. The upper leg, hips, and shoulders were stabilised with safety belts. Following one submaximal trial contraction, three maximal isometric knee-extensions and flexions (5 s) at knee angles of 90° were performed. Maximal contractions were interspersed by 40-second rest intervals. The highest isometric extension torques (Nm) of the manually smoothed curves were selected as maximal isometric torque. For each variable, legs of persons were allocated to groups of weakest and strongest leg.

### 2.5. Statistical Analysis

All calculations were performed using the Statistical Package for the Social Sciences 18.0 (SPSS). Data are expressed as means ± SD. Comparisons (for Borg ratings of perceived exertion) between different observations within the same subjects were made by paired-sample *t*-tests. Univariate relationships between parameters were examined by Pearson correlation coefficients, but for EDSS relations were examined by Spearman correlations. In these analyses (relations between 12 parameters were studied), Bonferroni corrections were applied (level of significance was set at *P* ≤ 0.004). Multivariate regression analyses were separately applied to examine relations between 6-minute walking distance (as a percentage of normal value) and EDSS score, muscle strength, and MRT. Because walking capacity was already normalized for age, gender, body weight, and height, these factors were not included in this model. In total, four models were created in which a different outcome from the strength test in subgroups of strongest or weakest leg was included for knee flexors and extensors. In the four different regression models, muscle strength of the weakest and strongest leg, both for flexion and extension, is included separately (dependent variable), while MRT and EDSS (dependent variables) and walking capacity (independent variable) are always the same per person. Statistical significance was set at *P* < 0.05 (2-tailed).

## 3. Results

### 3.1. Subject Characteristics

Twenty-four MS patients were included in this study (see Table 1). The following medications were prescribed to the subjects: glatiramer acetate (*n* = 3), natalizumab (*n* = 5), interferons (*n* = 11), muscle relaxing drugs (*n* = 1), analgesics (*n* = 5), and nonsteroid anti-inflammatory drugs (*n* = 1).

### 3.2. Exercise and Walking Test Data

During the cycling exercise test (see Table 2), an exercise intensity of 66 ± 8% of predicted maximal heart rate was elicited (corresponding to a blood lactate content of 3.0 ± 0.8 mmol/L and Borg ratings of perceived exertion of 12 ± 1). It is thus observed that a low-to-moderate exercise intensity was elicited during this cycling test. A MRT of 45 ± 16 seconds was achieved. During the 6-minute walking test, subjects walked a total distance of 529 ± 108 m with a perceived physical effort that was significantly higher (Borg ratings of perceived exertion 13 ± 2) as opposed to the cycling test (*P* < 0.05). During the strength test, knee extension peak torques of 139 ± 50 and 114 ± 41 Nm, in the strongest and weakest leg, respectively, were achieved. Knee flexion peak torques of 56  ±  19 and 44  ±  20 Nm, in the strongest and weakest leg, respectively, were achieved.

### 3.3. Correlations

Walking capacity correlated only with EDSS score (*r* = −0.70, *P* ≤ 0.004). Subject characteristics (age, body weight and height, body composition, and physical activity), muscle strength, and MRT did not correlate with walking capacity. Significant correlations were found between knee extension peak torque (of weakest and strongest leg) and body weight (*r* = 0.63, *P* ≤ 0.004), body height (*r* = 0.58–0.67, *P* ≤ 0.004), and leg lean tissue mass (*r* = 0.68–0.77, *P* ≤ 0.004).

### 3.4. Regression Analysis

In Table 3, regression models predicting walking capacity (as a percentage of normal value) are displayed. In all models EDSS score was independently related to walking capacity (*P* < 0.05). MRT was independently related to walking distance (*P* < 0.05) in both models where knee extensor torque was assessed and in the model where knee flexor torque of the weakest leg was assessed. In the model in which knee flexion peak torque of weakest leg was assessed, MRT showed a trend towards a relationship with walking capacity (*P* = 0.052).

## 4. Discussion

In this pilot study, we discriminated between the impact of maximal muscle strength versus muscle oxidative capacity on walking capacity in subjects with multiple sclerosis (MS). We found that, next to expanded disability status score (EDSS), 6-minute walking distance (as a percentage of normal value) was primarily related to muscle oxidative capacity and not to maximal muscle strength.

According to clinical guidelines, it is agreed that when a single measurement of walking capacity is executed in a patient, the percentage of predicted normal value should be used [10]. Because subject characteristics (age, gender, body weight, and height) might affect walking distance (in meters), it is important to correct for these variables to obtain a valid reflection of walking capacity. This methodological choice led to the conclusion that, next to overall disability level, muscle oxidative capacity is of greater impact on walking capacity in subjects with MS as opposed to maximal muscle strength. However, it should be mentioned that walking capacity in patients with MS is also dependent on the severity of neurologic symptoms. It thus follows that even though skeletal muscle oxidative capacity is more related to walking capacity, as opposed to muscle strength, it only partly explains the walking capacity.

It might be suggested that primarily muscle oxidative capacity should be targeted during exercise intervention in subjects with MS. Current literature seems to support this assumption. A recent meta-analysis evaluated the impact of strength training on walking capacity in subjects with MS, and found that although muscle strength improved significantly as result of strength training intervention, walking capacity was not always affected [31]. The authors argue that a lack of a significant impact of resistance training on walking capacity might be due to low study sample sizes, a possible ceiling effect with regard to walking capacity (when using short walking tests), a different selection of strength training modalities (intensity, number of contractions and/or series), and/or a short duration of exercise interventions [31]. It follows that the current literature cannot finally confirm that strength training interventions indeed are effective to increase walking capacity in subjects with MS. Endurance training and/or treadmill training, on the other hand, has almost consistently been shown to improve walking capacity in subjects with MS. Based on a recent systematic review on treadmill training [32] and on individual studies [33–36], nearly always significant improvements in walking capacity as result of this type of exercise training have been found. It could be hypothesized that endurance training interventions could be of greater impact on walking capacity, when opposed to strength training interventions in subjects with MS. Sebapathy and colleagues have compared the impact of eight weeks of resistance or endurance training in MS patients but did not observe a significantly different change in walking capacity as result of different interventions [37]. However, this study was limited by a relatively low sample size (16 participants) and short intervention period so more studies should be implemented to directly compare the impact of strength versus endurance exercise training on walking capacity.

In all regression models EDSS score was a significant predictor of walking capacity. Moreover, EDSS score turned out to be the best predictor of walking capacity. Because EDSS score in patients with MS is primarily not only based on walking distance in daily life but also is based on other anomalies in functional systems that affect walking capacity (balance, vision, and so forth), this might explain the high predictability of this parameter. However, it is important to take the level of disability into account when examining the impact of maximal muscle strength versus oxidative capacity on walking capacity in subjects with MS.

In the present study, patients with a relatively short disease duration, low EDSS, and few with a progressive type of MS were studied. It thus follows that results from the present study only apply to patients with such clinical profile. Moreover, the study sample size was small. Additional studies remain needed to examine the impact of muscle strength versus muscle oxidative capacity on walking capacity in large cohorts of MS patients with higher level of disability.

Because the physical tests were executed on separate days, the outcomes from these tests were not affected by the development of fatigue as it would occur when executing different tests on the same day. On the other hand, it should be mentioned that the patients' physical performance, neurologic symptoms, and/or fatigue could have slightly differed between these test days.

This study might be limited by a lack of measurement of plantar and hip flexor strength, which might also be related to walking capacity.

In conclusion, walking capacity is more related to muscle oxidative capacity than maximal muscle strength, not taking neurologic symptoms and deficits into account. Additional study is needed to further examine/verify these findings.

## Figures and Tables

**Table 1:** Subject characteristics.

	Means ± SD (or number)
Age	47 ± 11
Gender (*n* male)	12 (50%)
Height (cm)	170 ± 18
Weight (kg)	74 ± 15
Body mass index (kg/m²)	25.1 ± 4.5
Adipose tissue mass (kg)	24.0 ± 9.1
Lean tissue mass (kg)	42.6 ± 9.5
% Adipose tissue mass	35.6 ± 8.6
Physical activity (MET/h/week)	16 ± 10
Expanded disability status scale	3.1 ± 1.2
MS type (*n*)	
SPMS	9
RRMS	13
PPMS	1
PRMS	1
Disease duration (years)	11 ± 8
Modified fatigue impact scale (total score)	42 ± 18
Multiple sclerosis functional composite (total score)	42 ± 19

SPMS: secondary progressive multiple sclerosis; RRMS: relapsing remitting multiple sclerosis; PPMS: primary progressive multiple sclerosis; PRMS: progressive relapsing multiple sclerosis.

**Table 2:** Exercise testing.

	Means ± SD
Cycling test	
Cycling power output (W)	44 ± 16
Resting heart rate (bts/min)	82 ± 11
Resting oxygen uptake (mL/min)	300 ± 95
Cycling heart rate (bts/min)	114 ± 18
Cycling heart rate (% predicted maximum)	66 ± 8
Cycling oxygen uptake (mL/min)	1042 ± 267
Cycling expiratory volume (L/min)	29 ± 8
Cycling blood lactate content (mmol/L)	3.0 ± 0.8
Cycling Borg ratings of perceived exertion	12 ± 1
Oxygen deficit (mL)	554 ± 242
Mean response time (seconds)	45 ± 16
Strength test	
Extension peak torque strongest leg (Nm)	139 ± 50
Extension peak torque weakest leg (Nm)	114 ± 41
Flexion peak torque strongest leg (Nm)	56 ± 19
Flexion peak torque weakest leg (Nm)	44 ± 20
6-minute walking test	
Distance (meters)	529 ± 108
Borg ratings of perceived exertion	13 ± 2
Percentage of normal value (%)	86 ± 18

**Table 3:** Regression analyses for the prediction of 6-minute walking distance as a percentage of normal value.

	Standardized coefficient beta	*t* (*P* value)
Model 1: Knee extension, strongest leg		
Expanded disability status scale*	−0.72	−4.78 (<0.001)
Mean response time*	0.33	2.20 (<0.05)
Knee extension peak torque strongest leg	0.09	0.63 (0.53)
Model 2: Knee extension, weakest leg		
Expanded disability status scale*	−0.69	−4.41 (<0.001)
Mean response time*	0.33	2.20 (<0.05)
Knee extension peak torque weakest leg	0.13	0.82 (0.42)
Model 3: Knee flexion, strongest leg		
Expanded disability status scale*	−0.73	−4.77 (<0.001)
Mean response time*	0.32	2.12 (<0.05)
Knee flexion peak torque strongest leg	−0.03	−0.17 (0.87)
Model 4: Knee flexion, weakest leg		
Expanded disability status scale*	−0.69	−4.37 (<0.001)
Mean response time	0.31	2.08 (0.05)
Knee flexion peak torque weakest leg	0.11	0.70 (0.49)

*P < 0.05.
